# COVID-19 Does Not Increase the Risk of Spontaneous Cervical Artery Dissection

**DOI:** 10.7759/cureus.47524

**Published:** 2023-10-23

**Authors:** Robert J Trager, Zachary A Cupler, Elainie C Theodorou, Jeffery A Dusek

**Affiliations:** 1 Department of Chiropractic, Connor Whole Health, University Hospitals Cleveland Medical Center, Cleveland, USA; 2 Department of Family Medicine and Community Health, School of Medicine, Case Western Reserve University, Cleveland, USA; 3 Department of Biostatistics and Bioinformatics, Clinical Research Training Program, Duke University School of Medicine, Durham, USA; 4 Physical Medicine & Rehabilitative Services, Butler VA (Veterans Affairs) Health Care System, Butler, USA; 5 Institute for Clinical Research Education, University of Pittsburgh School of Medicine, Pittsburgh, USA; 6 Science Research and Engineering Program, Hathaway Brown School, Cleveland, USA

**Keywords:** coronavirus infections, respiratory infections, covid-19, internal carotid artery dissection, vertebral artery dissection

## Abstract

Background

Case reports have raised the possibility of an association between coronavirus disease 2019 (COVID-19) and spontaneous cervical artery dissection (sCeAD), yet no large studies have examined this association. We hypothesized that adults with confirmed COVID-19 would have an increased risk of sCeAD over the subsequent six months compared to test-negative controls after adjusting for confounding variables.

Methods

We obtained data from a United States medical records network (TriNetX, Inc., Cambridge, MA) of >106 million patients, providing adequate power needed for this rare outcome. We identified two cohorts of adults meeting the criteria of (1) test-confirmed COVID-19 or (2) non-COVID-19 test-negative controls, from April 1, 2020, to December 31, 2022. Patients with previous COVID-19 or conditions predisposing to sCeAD were excluded. Propensity matching was used to control for variables associated with sCeAD and markers of healthcare utilization.

Results

The number of patients reduced from before matching (COVID-19: 491,592; non-COVID-19: 1,472,895) to after matching, resulting in 491,115 patients per cohort. After matching, there were 22 cases of sCeAD in the COVID-19 cohort (0.0045%) and 20 cases in the non-COVID-19 cohort (0.0041%), yielding a risk ratio of 1.10 (95% CI: 0.60-2.02; P* *= 0.7576). Both cohorts had a median of five healthcare visits during follow-up.

Conclusions

Our results suggest that COVID-19 is not a risk factor for sCeAD. This null finding alleviates the concern raised by initial case reports and may better direct future research efforts on this topic.

## Introduction

A cervical artery dissection is a tear and hematoma of the arterial wall affecting either one of the paired carotid arteries in the anterior neck or paired vertebral arteries in the posterior neck [1]. In the absence of trauma, this condition is referred to as spontaneous cervical artery dissection (sCeAD) [1]. sCeAD has an incidence rate of nine per 100,000 person-years [2]. Patients experiencing a sCeAD often present with a primary complaint of headache or neck pain, yet may progress to develop a stroke within hours or days [1]. To date, there is a limited understanding of the risk factors for sCeAD [1]. Awareness of the prodromal signs, symptoms, and risk factors for sCeAD is needed to help clinicians recognize patients with this evolving condition early and refer them for emergent medical management [1].

Several cases of sCeAD during or following severe acute respiratory syndrome coronavirus 2 (SARS‑CoV‑2), also called coronavirus disease 2019 (COVID-19), have been described in the literature [3-7]. COVID-19 is a known risk factor for ischemic stroke [8] and is suspected of increasing the likelihood of vasculitis and dissections of other arteries, including the aorta and coronary arteries [3]. While evidence is lacking, authors have speculated that COVID-19 might cause sCeAD via an exaggerated systemic inflammatory response, leading to endothelial dysfunction, or by directly invading vascular endothelial cells via their surface angiotensin-converting enzyme 2 receptors and causing endothelial damage [4,5,7].

Aside from COVID-19, multiple studies have found an association between respiratory tract infections and sCeAD [9-12], a finding which remains even after accounting for potential mild neck trauma related to coughing, sneezing, and vomiting [9]. An increased risk of sCeAD after influenza-like illnesses has been found to last up to three months [11,12]. Patients with sCeAD have increased white blood cell counts and a reduced activated partial thromboplastin time, signs which may reflect the underlying inflammation and hypercoagulable state that accompanies an infection [13,14]. It also remains possible that other non-respiratory infections increase the risk of sCeAD; however, this research remains limited [9]. The general relationship between respiratory infection and sCeAD identified in prior studies provided the rationale to examine if COVID-19, a unique and widespread respiratory illness, could demonstrate a similar association with sCeAD risk [9-12].

Although case reports have proposed a potential association between COVID-19 and sCeAD, this has remained unexplored by larger study designs. We aim to examine this possible association using large United States (US) cohorts, with the hypothesis that adults with confirmed COVID-19 will have an increased risk of sCeAD over the subsequent six months compared to test-negative controls.

## Materials and methods

Study design

We used a retrospective cohort study design with active comparator and new user features to reduce bias, which we registered a priori [15]. We included patients meeting criteria starting April 1, 2020, coinciding with the availability of specific COVID-19 codes, until December 31, 2022, to allow for ascertainment of the outcome of interest, sCeAD, during follow-up prior to the query date (July 21, 2023). This study was determined "Not Human Subjects" by the University Hospitals Institutional Review Board (Cleveland, Ohio, USA; STUDY20230352).

Setting and data source

This study used data from the US TriNetX research network (TriNetX Inc., Cambridge, MA), which includes linked, aggregated, de-identified medical records, medical claims, and pharmaceutical claims data. TriNetX includes routinely collected data from over 106 million patients attending 76 healthcare institutions relating to patient care, diagnoses, and test results, and includes both insured and uninsured patients. The dataset may be searched using standardized coding systems such as the International Classification of Diseases, 10th Edition (ICD-10). With regards to COVID-19, the data are harmonized and must meet several requirements, including standardization of coding of COVID-19 testing data and at least 80% data completeness for demographics [16].

Participants

We included individuals of at least 18 years of age who underwent COVID-19 testing. To optimize the accuracy of baseline medical records with respect to sCeAD risk and ensure patients were actively engaged in the healthcare system, we required patients to have any healthcare visit and blood pressure measurement between one month and two years preceding the index date of their COVID-19 test. To limit loss to follow-up, ensure greater validity of our test-negative case definition, and limit crossover of test-negative controls, we required that all patients had at least one healthcare visit within six months following the index COVID-19 testing date.

The COVID-19 cohort included adults at their first instance of a confirmed clinical diagnosis of COVID-19 illness (ICD-10: U07.1) or positive ribonucleic acid (RNA) COVID-19 test result occurring in the dataset, using a custom curated TriNetX code, which includes several RNA-based COVID-19 testing methods (TriNetX: 9088, “SARS coronavirus 2 and related RNA”). The active control non-COVID-19 cohort consisted of adults included at the first instance of a negative RNA COVID-19 test (TriNetX: 9088). It is important to clarify that we did not use COVID-19 antibody tests to distinguish between cohorts, since antibodies may arise as the result of vaccination rather than illness and variable access to antibody testing.

We excluded patients with a previous diagnosis of cerebrovascular disease, such as hemorrhage, infarction, and occlusion or stenosis of the cerebral or cervical arteries, and those with previous dissection or aneurysm of the carotid, vertebral, or basilar arteries, or aorta (Appendix 1: Exclusion codes) [17]. We excluded patients with hereditary conditions that predispose to sCeAD such as Alport syndrome, arterial tortuosity syndrome, fibromuscular dysplasia, Ehlers-Danlos syndrome, Loeys-Dietz syndrome, Marfan syndrome, Eagle syndrome, and osteogenesis imperfecta [17]. To help ensure dissections were spontaneous, we excluded those with external causes of morbidity (e.g., motor vehicle accidents and falls) and preceding head or neck trauma [17]. We excluded individuals with bacterial pneumonia or influenza in the three months preceding and including the date of COVID-19 testing, considering the evidence that influenza-like illness increases the risk of sCeAD [9-13]. Via these exclusions, we sought to minimize confounding related to other recent or concurrent respiratory infections. Patients who developed COVID-19 during follow-up were excluded from the non-COVID-19 cohort.

To account for test selection bias and the potential simultaneity of COVID-19 and sCeAD due to universal COVID-19 testing in acute care settings, we excluded patients registering a positive or negative COVID-19 test on the same date of an inpatient, critical care, hospital observation, or emergency visit. As a related exclusion, we began the follow-up window the day after COVID-19 testing. This helped ensure patients’ medical records were accurate at the time of the index testing date.

Variables

We used propensity matching to balance confounding variables between cohorts and reduce bias. Variables present within 365 days preceding and including the index date of COVID-19 testing were eligible for matching. Variables were selected for matching based chiefly on a positive or negative association with sCeAD, including (Appendix 2: Propensity-matched variables) adverse socioeconomic factors (i.e., problems related to education, employment, and housing/income; negative) [9], body mass index (negative) [17], demographics (age, sex, race/ethnicity; positive or negative) [17], hypertension (negative) [18], head and neck imaging (positive), migraines (positive) [17], and pregnancy (positive) [19]. To further control for potential cardiovascular risk factors, we matched for cardiovascular medications (e.g., antihypertensives) and cardiovascular procedures (e.g., cardiography and cardiovascular monitoring). Considering the potential for sCeAD to remain undiagnosed during the pandemic in relation to healthcare underutilization [20,21], we matched for substance use disorders, emergency department and inpatient visits, and hospital observation services. We also controlled for COVID-19 vaccination. Diabetes, hyperlipidemia, and smoking were not propensity-matched, considering a recent systematic review found no association between these conditions and sCeAD [18].

We examined for the occurrence of any sCeAD from the day after the index date of COVID-19 testing through the following six months, based on previous research showing that vascular endothelial dysfunction may persist amongst those with COVID-19 for six months [22]. ICD-10 codes were used to identify any occurrence of either carotid artery dissection (I77.71) or vertebral artery dissection (I77.74). Considering that sCeAD is rare, and each type of dissection has similar risk factors, these two etiologies of sCeAD were pooled as a single outcome. We did not examine stroke as an outcome, which has different predisposing factors [23] and would require different selection criteria and propensity-matched variables.

Statistical methods

We compared between-cohort baseline characteristics using a Pearson chi-squared test or independent samples t-test. Standardized mean differences (SMD) > 0.1 represented residual imbalance. We used logistic regression to calculate propensity scores, matching with a 1:1 ratio, greedy-nearest neighbor algorithm, and caliper width of 0.1 pooled standard deviations (SDs). Risk ratios (RRs) were calculated by dividing the incidence proportion of sCeAD per cohort (i.e., COVID-19 divided by non-COVID-19). Significance was evaluated at p < 0.05. We did not make imputations for missing data. We plotted cumulative incidence and propensity score density using R version 4.2.2 (R Foundation for Statistical Computing, Vienna, Austria) and the ggplot2 package.

As a sensitivity analysis, we examined time-to-event data to better understand the timing of sCeAD throughout the follow-up period. We graphed the cumulative incidence of sCeAD per cohort over the six-month window, following propensity matching, to identify any inflection points that may have corresponded to periods of higher or lower incidence of sCeAD. For another sensitivity analysis, we examined the instances of any healthcare visit during follow-up after propensity matching as a metric of healthcare utilization. We compared the mean and median number of visits per patient per cohort and corresponding standard deviations.

Considering there was a limited amount of data regarding sCeAD and COVID-19, we used epidemiological data regarding sCeAD to estimate a sample size [2]. We calculated a total required sample size of 363,822 via G*Power (version 3.1.9.7, Kiel University, Kiel, Germany) using a z-test to examine a difference in incidence proportion between cohorts (0.002% vs. 0.012%) using an alpha error of 0.05, two tails, allocation ratio of one, and power of 0.95.

## Results

Participants

We identified patients from approximately 50 healthcare organizations per cohort (COVID-19: 58; non-COVID-19: 51). Before propensity matching, there were 491,592 patients in the COVID-19 cohort and 1,472,895 patients in the non-COVID-19 cohort. After matching, there were 491,115 patients per cohort (mean age = 49.2, SD = 17.2 years, 64% female). Before matching, the COVID-19 cohort had a lower frequency of patients with cardiovascular medications and anti-hypertensives (SMD > 0.1; Table 1). After matching, there were no meaningful between-cohort differences for any matched covariate (SMD < 0.1; Table 1).

**Table 1:** Baseline cohort characteristics before and after propensity score matching * Data used for our primary outcome; COVID-19: coronavirus disease 2019; SD: standard deviation; SMD: standardized mean difference.

Variable	Before matching			After matching*		
	COVID-19	Non-COVID-19	SMD	COVID-19	Non-COVID-19	SMD
N	491,592	1,472,895		491,115	491,115	
Age at index (SD)	49.2 (17.2)	50.9 (17.5)	0.094	49.2 (17.2)	49.4 (17.0)	0.009
Age (min-max)	18-90	19-90		18-90	19-90	
Sex						
Female	315,090 (64%)	916,530 (62%)	0.039	314,803 (64%)	313,360 (64%)	0.006
Male	176,410 (36%)	556,120 (38%)	0.039	176,220 (36%)	177,676 (36%)	0.006
Body mass index (SD)	30.4 (6.9)	29.8 (6.8)	0.080	30.4 (6.9)	29.9 (6.9)	0.073
Race/ethnicity						
Asian	11,825 (2%)	33,388 (2%)	0.009	11,804 (2%)	11,777 (2%)	<0.001
Black or African American	75,864 (15%)	248,278 (17%)	0.039	75,817 (15%)	76,519 (16%)	0.004
Hispanic or Latino	37,072 (8%)	92,562 (6%)	0.050	36,975 (8%)	36,171 (7%)	0.006
Not Hispanic or Latino	334,663 (68%)	966,620 (66%)	0.052	334,288 (68%)	334,195 (68%)	<0.001
White	341,703 (70%)	1,026,148 (70%)	0.003	341,363 (70%)	340,869 (69%)	0.002
Diagnoses						
Adverse socioeconomic and psychosocial circumstances	14,184 (3%)	34,748 (2%)	0.033	14,139 (3%)	13,962 (3%)	0.002
Hypertensive diseases	153,329 (31%)	420,373 (29%)	0.058	152,883 (31%)	154,910 (32%)	0.009
Mental and behavioral disorders due to psychoactive substance use	39,618 (8%)	147,985 (10%)	0.069	39,615 (8%)	39,685 (8%)	0.001
Migraine	27,252 (6%)	66,905 (5%)	0.046	27,194 (6%)	26,600 (5%)	0.005
Pregnancy, childbirth, and puerperium	26,179 (5%)	79,399 (5%)	0.003	26,173 (5%)	25,773 (5%)	0.004
Procedures/tests/medications						
Antihypertensives	18,305 (4%)	99,985 (7%)	0.138	18,305 (4%)	17,335 (4%)	0.011
Cardiovascular medications	224,330 (46%)	842,836 (57%)	0.233	224,321 (46%)	225,150 (46%)	0.003
Cardiovascular procedures	101,308 (21%)	355,986 (24%)	0.086	101,201 (21%)	98,780 (20%)	0.012
COVID-19 vaccination, first dose	12,361 (3%)	21,275 (1%)	0.077	11,906 (2%)	10,558 (2%)	0.018
COVID-19 vaccination, second dose	10,294 (2%)	16,863 (1%)	0.075	9,836 (2%)	8,701 (2%)	0.017
Diagnostic imaging of head/neck	29,462 (6%)	99,118 (7%)	0.030	29,429 (6%)	28,135 (6%)	0.011
Visit: Emergency	98,356 (20%)	333,477 (23%)	0.064	98,316 (20%)	96,235 (20%)	0.011
Visit: Inpatient encounter	53,667 (11%)	193,829 (13%)	0.069	53,631 (11%)	49,716 (10%)	0.026

Descriptive data

**Figure 1:**
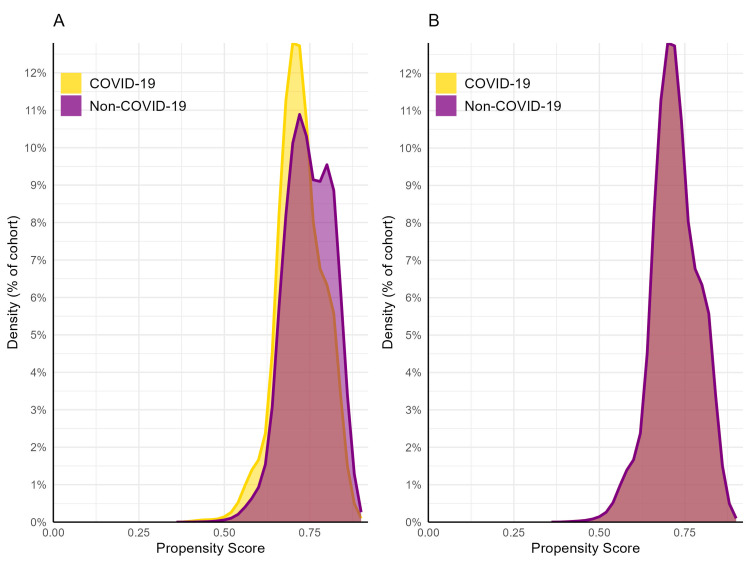
Propensity scores before (A) and after (B) matching The COVID-19 cohort is represented in gold while the non-COVID-19 cohort is purple. In image B, propensity score densities overlap indicating that the cohorts are well-matched following matching.

The mean number of data points was high in each cohort (COVID-19: 2,091; non-COVID-19: 2,114). After matching, the frequency of “unknown” demographic variables was similar between cohorts for unknown ethnicity (COVID-19: 24%, non-COVID-19: 25%; SMD = 0.004), unknown race (12% per cohort; SMD = 0.001), unknown sex (0% per cohort), and unknown age (0% per cohort). A graph of the propensity score densities revealed that the propensity scores were sufficiently matched (Figure 1). These findings indicated that there were no meaningful between-cohort differences regarding data completeness or residual covariate imbalance.

Key results

There was no significant difference in the risk of sCeAD over six months of follow-up from the index date of COVID-19 testing between cohorts both before and after propensity matching (Table 2). After matching, the incidence proportion of sCeAD in the COVID-19 cohort was 0.0045% in the COVID-19 cohort and 0.0041% in the non-COVID-19 cohort, yielding an RR of 1.10 (0.60-2.02; P = 0.7576).

**Table 2:** Association between sCeAD and COVID-19 * Data used for our primary outcome; COVID-19: coronavirus disease 2019; RR: risk ratio; CI: 95% confidence intervals; sCeAD: spontaneous cervical artery dissection.

	Before matching		After matching*	
	COVID-19, n = 491,592	Non-COVID-19, n = 1,472,895	COVID-19, n = 491,115	Non-COVID-19, n = 491,115
sCeAD, No. (%)	22 (0.0045)	72 (0.0049)	22 (0.0045%)	20 (0.0041%)
RR (CI)	0.92 (0.57-1.50; P = 0.7266)	(Reference)	1.10 (0.60-2.02; P = 0.7576)	(Reference)

Sensitivity analyses

**Figure 2:**
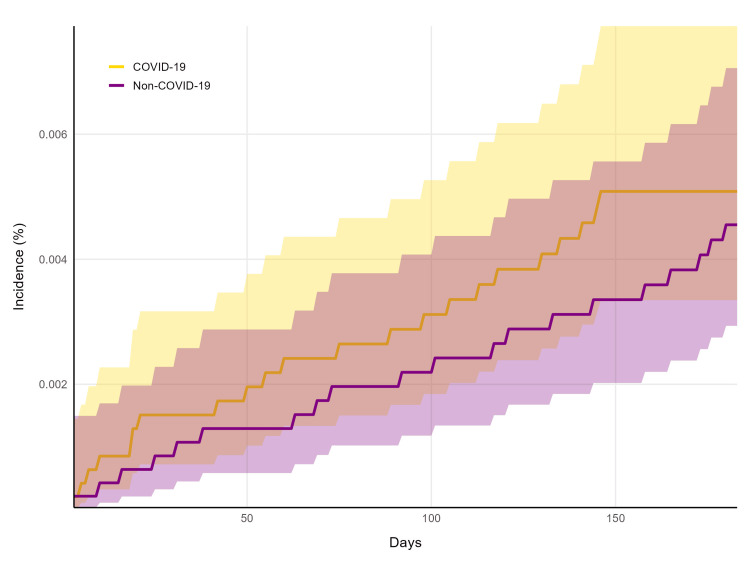
Cumulative incidence of sCeAD The incidences of spontaneous cervical artery dissection (sCeAD) in the coronavirus disease 2019 (COVID-19) cohort (gold) and non-COVID-19 cohort (purple) are shown over the six-month follow-up window (183 days). Shaded regions indicate 95% confidence intervals.

A cumulative incidence graph indicated that the incidence of sCeAD was similar between cohorts throughout follow-up, without any clear increase or decrease in risk between cohorts at any time point (Figure 2). The 95% confidence intervals overlapped throughout the entire period. After matching, the mean number of healthcare visits after the index date of COVID-19 testing was 7.7 (SD = 9.4) in the COVID-19 cohort and 8.0 (SD = 8.0) in the non-COVID-19 cohort (P < 0.0001), while both cohorts had an identical median of five follow-up visits over the six-month window after COVID-19 testing.

## Discussion

To our knowledge, the present study was the first to examine the potential association between COVID-19 and sCeAD. Furthermore, the study included a large total population of 982,230 patients. The study strengths include the use of a US national sample, propensity-matched control group, and test-negative design. Our study suggests that COVID-19 is not a significant risk factor for sCeAD up to six months after positive COVID-19 RNA test results. The cumulative incidence proportion over six months translates to an approximate incidence rate of nine per 100,000 person-years (COVID-19 cohort) and eight per 100,000 person-years (non-COVID-19 cohort), both of which are comparable to the expected incidence of sCeAD as described previously in the literature (i.e., nine per 100,000 person-years [2]). This serves as a marker of the validity of our findings and supports our impression that COVID-19 is not a risk factor for sCeAD.

While our findings are discordant and refute previous case reports highlighting a temporal association between COVID-19 and sCeAD [3-7], these cases may have identified coincidental occurrences of sCeAD among a backdrop of a high number of COVID-19 cases and potentially co-existing non-COVID-19 respiratory tract infections. Case reports have publication bias due to their small sample size and lack of a control group, while the current study was designed to overcome these limitations.

While the COVID-19 cohort had a significantly lower number of mean follow-up healthcare visits compared to the non-COVID-19 cohort, the absolute between-cohort difference was less than one visit, and both cohorts had a median of five follow-up visits. In addition, the relatively high number of mean follow-up visits in each cohort (> nine over a six-month window) suggests that our analysis included patients who were actively engaged in the healthcare system rather than those individuals who remained at home and did not present for health care. This is a marker of the success of our strategy to require patients to have preceding/follow-up visit(s) and propensity match for measures of healthcare utilization. Overall, we suggest that our findings are unexplained by between-cohort differences in healthcare utilization.

There are several explanations as to why COVID-19 would not increase the risk of sCeAD. In general, sCeAD is complex and multifactorial [17], and several risk factors identified in smaller studies were shown to not be significant in a recent systematic review (e.g., diabetes, smoking) [18]. However, it is possible that patients diagnosed with COVID-19 diminished their activity levels and avoided activities that could otherwise increase the risk of sCeAD (e.g., minor trauma related to sports) [17]. While COVID-19 does cause a hypercoagulable state, it is possible that hypercoagulation alone is insufficient to significantly elevate the risk of sCeAD. A recent meta-analysis found that diabetes and hyperlipidemia, which are known to promote hypercoagulation, do not increase the risk of sCeAD [18].

We suggest that excluding predisposing conditions such as Ehlers-Danlos syndrome would not have affected our findings, considering the comparable incidence of sCeAD in our study to the known literature (e.g., 8-9/100,000 person-years) [2]. In addition, none of the previous case reports identifying a temporal association between COVID-19 and sCeAD mentioned underlying connective tissue disorders as a comorbid risk factor [3-7]. Other hypotheses for an association between COVID-19 vaccination and sCeAD [24] or COVID-19 oropharyngeal swabbing (e.g., causing neck hyperextension) and sCeAD [25] appear unlikely given our findings of a typical, expected incidence rate of sCeAD for the general population in both cohorts who had an equal exposure to both vaccination and COVID-19 testing.

One study from Austria reported a reduction in cases of sCeAD during the COVID-19 pandemic compared to preceding the pandemic [20]. This led the authors to suggest that public health measures during the pandemic led to a reduction in sCeAD cases (i.e., masking mandates could have decreased the incidence of other upper respiratory infections, which may increase the risk of sCeAD). Other research suggested that emergency department visits decreased during the pandemic [21], thereby raising the possibility of reduced detection of sCeAD amongst those with COVID-19 illness. While pandemic-related restrictions are a potential unmeasured confounder in our study, our method of excluding patients with other respiratory infections at baseline and controlling for markers of healthcare utilization aimed to diminish any such influence from these factors.

It remains unclear why other respiratory infections such as influenza-like illness have been found to increase the risk of sCeAD [11,12], while our study found no association between COVID-19 and sCeAD. Regardless, while inflammation, minor trauma (e.g., coughing/sneezing), and hypercoagulation have been suspected to account for the association between respiratory infection and sCeAD, the mechanism remains unclear [9,12]. In addition, it remains possible that medications used to treat respiratory infections are the responsible etiologic agent, rather than the infections themselves. Chiefly, non-COVID-19 respiratory infections are often treated with fluoroquinolones, which some evidence suggests independently increases the risk of sCeAD [26]. While beyond the scope of the current study, further research may be needed to clarify whether associations between upper respiratory infections and sCeAD directly result from the infections or are an adverse sequela of the medications used to treat the infections.

While there is some overlap between the risk factors for stroke and sCeAD (e.g., hypertension), it appears that COVID-19 specifically increases the risk of ischemic stroke [8] but not sCeAD. This may relate to differences in the predisposing factors for each condition. While ischemic stroke is more strongly associated with diabetes and hyperlipidemia, these comorbidities are not significant risk factors for sCeAD [18,23].

Limitations

As an observational study, there is a possibility of unmeasured confounding due to the variables and constraints of the study dataset. We were unable to control for the known variants of COVID-19 (e.g., omicron, alpha, and delta), COVID-19 disease severity, and had a limited ability to control for social determinants of health and genetic variants related to sCeAD and non-COVID-19 respiratory infections. It was impractical to exclude individuals with respiratory infections relating to an unspecified organism, or those with acute respiratory infections such as the common cold. In addition, viral respiratory infections are often not recorded as patients may not seek medical attention [12]. We were also unable to control for secondary respiratory infections that may have developed later in the course of COVID-19 illness. Despite our rigorous test-negative design, there are potential sources of misclassification. As RNA-based COVID-19 tests have a false positive rate of 0.05 and a false negative rate of 0.1 to 0.3 [27], initial testing could occasionally be inaccurate. However, bias related to initial false positive or negative results was minimized via selection criteria and requiring a healthcare visit during follow-up. One scenario that was difficult to account for was patients who could have tested negative for COVID-19, then contracted COVID-19, but did not undergo confirmatory testing despite having a required follow-up visit. Study results may only be generalizable to the US and may not apply to other countries, which may have a different baseline susceptibility to sCeAD, different COVID-19 prevalence, or dissimilar public health measures. Study findings may also not apply to future COVID-19 variants.

## Conclusions

Our study involving over 982,230 matched US adults provides robust evidence that COVID-19 is not a meaningful risk factor for sCeAD. These findings were not likely explained by differential healthcare utilization or other confounding variables. Despite previous case reports suggesting a temporal association between COVID-19 and sCeAD, our findings indicate that these cases may represent a coincidental rather than a causal relationship. Further research is needed to better clarify the association between other respiratory infections, sCeAD, and intervening medications.

## Appendices

Appendix 1: Exclusion codes

**Table 3:** Exclusion codes COVID-19: coronavirus disease 2019; LOINC: Logical Observation Identifiers Names and Codes; NAA: nucleic acid amplification; RNA: ribonucleic acid; SARS‑CoV‑2: severe acute respiratory syndrome coronavirus 2; SNOMED: Systemized Nomenclature of Medicine; -∞: exclusions over any time available in the preceding record; *: exclusions also apply to the testing date and following six months after in the non-COVID-19 cohort; TNX: curated TriNetX code including several positive tests for COVID-19; ICD-10: International Classification of Diseases, 10th Edition.

Exclusions	Definition	Duration (days)
Diagnoses (ICD-10)		
B34.2	Coronavirus infection, unspecified	-∞, -1*
B97.29	Other coronavirus infections, classified elsewhere	-∞, -1*
U07.1	COVID-19, virus identified	-∞, -1*
U07.2	COVID-19, virus not identified	-∞, -1*
U09	Post COVID-19 condition	-∞, -1*
Z86.16	Personal history of COVID-19	-∞, -1*
G45	Transient ischemic attacks and related syndromes	-∞, 0
G46	Vascular syndromes of the brain in cerebrovascular diseases	-∞, 0
I60-I69	Cerebrovascular diseases	-∞, 0
I71	Aortic aneurysm and dissection	-∞, 0
I72.0	Aneurysm of carotid artery	-∞, 0
I72.6	Aneurysm of vertebral artery	-∞, 0
Z86.73	Personal history of transient ischemic attack, and cerebral infarction without residual deficits	-∞, 0
I77.7	Other arterial dissection (including carotid, vertebral, basilar, and others)	-∞, 0
I77.81	Aortic ectasia	-∞, 0
M85.88	Eagle syndrome	-∞, 183
Q28	Other congenital malformations of circulatory system (e.g., precerebral)	-∞, 183
Q78.0	Osteogenesis imperfecta	-∞, 183
Q79.6	Ehlers-Danlos syndromes	-∞, 183
Q87.4	Marfan syndrome	-∞, 183
Q87.81	Alport syndrome	-∞, 183
Q87.89	Loeys-Dietz syndrome	-∞, 183
I77.3	Arterial fibromuscular dysplasia	-∞, 183
J09	Influenza due to certain identified influenza viruses	-91, 0
J10	Influenza due to other identified influenza virus	-91, 0
J13	Pneumonia due to Streptococcus pneumoniae	-91, 0
J14	Pneumonia due to Haemophilus influenzae	-91, 0
J15	Bacterial pneumonia, not elsewhere classified	-91, 0
S00-S09	Injuries to the head	-30, 0
S10-S19	Injuries to the neck (including arteries)	-30, 0
V00-Y99	External causes of morbidity (e.g., motor vehicle accidents and falls)	-30, 183
Procedures		
35301	Thromboendarterectomy; carotid, vertebral, subclavian, by neck incision (CPT)	-∞, 0
1022228	Transcatheter placement of intravascular stent(s), cervical carotid artery (CPT)	-∞, 0
0076T	Transcatheter placement of extracranial vertebral artery stent(s) (CPT)	-∞, 0
118807008	Procedure on the artery of the head or neck (SNOMED)	-∞, 0
Laboratory tests (LOINC)		
94763-0	SARS-CoV-2 (COVID-19) {presence}, specimen, organism-specific culture	-∞, -1*
9088^TNX^	SARS-CoV-2 and related RNA {presence}	-∞, -1*
40982-1	Influenza B RNA {presence}, specimen by NAA, probe detection	-91, 0
34487-9	Influenza A RNA {presence}, specimen by NAA, probe detection	-91, 0

Appendix 2: Propensity-matched variables

**Table 4:** Propensity-matched variables ATC: Anatomical Therapeutic Chemical Classification; COVID-19: coronavirus disease 2019; CPT: Current Procedural Terminology; ICD-10: International Classification of Diseases, 10th Edition; LOINC: Logical Observation Identifiers Names and Codes; TNX: custom TriNetX code; mRNA-LNP: messenger ribonucleic acid lipid nanoparticle; SARS-CoV-2: severe acute respiratory syndrome coronavirus 2; VANDF: Veterans Health Administration National Drug File.

Variable/code	Description
Demographics	Patient age, sex, race, ethnicity
Tests (LOINC)	
39156-5	Body mass index
Diagnoses (ICD-10)	
F10-F19	Mental and behavioral disorders due to psychoactive substance use
G43	Migraine
I10-I16	Hypertensive diseases
O00-O9A	Pregnancy, childbirth, and puerperium
Z55-Z65	Persons with potential health hazards related to socioeconomic and psychosocial circumstances
Medications	
CV000 (VANDF)	Cardiovascular medications
C02 (ATC)	Antihypertensives
Procedures	
0001A	Immunization administration by intramuscular injection of SARS-CoV-2 COVID-19 vaccine, mRNA-LNP, spike protein, preservative-free, 30 mcg/0.3mL dosage, diluent reconstituted; first dose
0002A	Immunization administration by intramuscular injection of SARS-CoV-2 COVID-19 vaccine, mRNA-LNP, spike protein, preservative-free, 30 mcg/0.3mL dosage, diluent reconstituted; second dose
1010253 (CPT)	Diagnostic radiology (diagnostic imaging) procedures of the head and neck
1012974 (CPT)	Cardiovascular procedures (e.g., cardiography and cardiovascular monitoring)
1013648 (CPT)	Hospital observation services
IMP Visit^TNX^	Inpatient encounter
EMER^TNX^	Emergency visit
